# Supplementary material for: Role and mechanism of NCAPD3 in promoting malignant behaviors in gastric cancer
Source: Front Pharmacol. 2024 Apr 22;15:1341039. doi: 10.3389/fphar.2024.1341039 (PMC11070777; doi:10.3389/fphar.2024.1341039)
Supplement: Supplementary file 11 [file DataSheet2.ZIP › GSEA/GO_CC/my_analysis.Gsea.1599461408869/GO_METHYLTRANSFERASE_COMPLEX.html]

Details for gene set GO\_METHYLTRANSFERASE\_COMPLEX[GSEA]

|  || Dataset | filtered\_dataset.sample\_info.cls#WT\_versus\_NCAPD3\_MUT |
| Phenotype | sample\_info.cls#WT\_versus\_NCAPD3\_MUT |
| Upregulated in class | WT |
| GeneSet | GO\_METHYLTRANSFERASE\_COMPLEX |
| Enrichment Score (ES) | 0.35589367 |
| Normalized Enrichment Score (NES) | 1.4638902 |
| Nominal p-value | 0.08983051 |
| FDR q-value | 0.6598619 |
| FWER p-Value | 0.99 |
Table: GSEA Results Summary

  

Fig 1: Enrichment plot: GO\_METHYLTRANSFERASE\_COMPLEX      
 Profile of the Running ES Score & Positions of GeneSet Members on the Rank Ordered List

  

| SYMBOL | TITLE | RANK IN GENE LIST | RANK METRIC SCORE | RUNNING ES | CORE ENRICHMENT || 1 | 23054 | NCOA6 | 77 | 0.838 | 0.0430 | Yes |
| 2 | 55006 | TRMT61B | 101 | 0.782 | 0.1181 | Yes |
| 3 | 152485 | ZNF827 | 268 | 0.618 | 0.0714 | Yes |
| 4 | 4297 | KMT2A | 337 | 0.573 | 0.0898 | Yes |
| 5 | 54554 | WDR5B | 395 | 0.539 | 0.1120 | Yes |
| 6 | 8607 | RUVBL1 | 451 | 0.506 | 0.1318 | Yes |
| 7 | 10785 | WDR4 | 465 | 0.495 | 0.1805 | Yes |
| 8 | 23468 | CBX5 | 484 | 0.485 | 0.2245 | Yes |
| 9 | 6632 | SNRPD1 | 563 | 0.440 | 0.2201 | Yes |
| 10 | 56339 | METTL3 | 613 | 0.417 | 0.2338 | Yes |
| 11 | 10445 | MCRS1 | 617 | 0.414 | 0.2801 | Yes |
| 12 | 22847 | ZNF507 | 618 | 0.413 | 0.3285 | Yes |
| 13 | 29890 | RBM15B | 651 | 0.400 | 0.3524 | Yes |
| 14 | 84148 | KAT8 | 757 | 0.344 | 0.3174 | Yes |
| 15 | 11091 | WDR5 | 822 | 0.304 | 0.3071 | Yes |
| 16 | 26121 | PRPF31 | 825 | 0.301 | 0.3409 | Yes |
| 17 | 10856 | RUVBL2 | 847 | 0.257 | 0.3559 | Yes |
| 18 | 2146 | EZH2 | 1001 | -0.406 | 0.2936 | No |
Table: GSEA details [plain text format]

  

Fig 2: GO\_METHYLTRANSFERASE\_COMPLEX      
 Blue-Pink O' Gram in the Space of the Analyzed GeneSet

  

Fig 3: GO\_METHYLTRANSFERASE\_COMPLEX: Random ES distribution      
 Gene set null distribution of ES for **GO\_METHYLTRANSFERASE\_COMPLEX**
